# PC-12 Cell Line as a Neuronal Cell Model for Biosensing Applications

**DOI:** 10.3390/bios12070500

**Published:** 2022-07-08

**Authors:** Daniela Oprea, Caroline G. Sanz, Madalina M. Barsan, Teodor Adrian Enache

**Affiliations:** National Institute of Materials Physics, Atomistilor 405A, 077125 Magurele, Romania; daniela.oprea@infim.ro (D.O.); caroline.sanz@infim.ro (C.G.S.); madalina.barsan@infim.ro (M.M.B.)

**Keywords:** PC12 cell line, biosensors, biosensing, analytical determination, neuronal stimulation, neurotransmitters, exocytosis, ion channel

## Abstract

PC-12 cells have been widely used as a neuronal line study model in many biosensing devices, mainly due to the neurogenic characteristics acquired after differentiation, such as high level of secreted neurotransmitter, neuron morphology characterized by neurite outgrowth, and expression of ion and neurotransmitter receptors. For understanding the pathophysiology processes involved in brain disorders, PC-12 cell line is extensively assessed in neuroscience research, including studies on neurotoxicity, neuroprotection, or neurosecretion. Various analytical technologies have been developed to investigate physicochemical processes and the biosensors based on optical and electrochemical techniques, among others, have been at the forefront of this development. This article summarizes the application of different biosensors in PC-12 cell cultures and presents the modern approaches employed in neuronal networks biosensing.

## 1. Introduction

In a normal, healthy, brain the neurons communicate with each other using electrical charges that travel down axons causing the release of chemicals and, through different pathways, perform every function of the brain such as sensations, movements, thoughts, memories, and feelings. The neurons are kept healthy by other cells of the brain, which are closely connected, and miscommunications in one area can disrupt other brain activities, meaning that brain disorders can result in widespread problems. In vitro assays are commonly employed for understanding the pathophysiology processes involved in brain disorders, thus decreasing the complexity of direct in vivo approaches [1].

In particular, primary neurons represent a powerful model to manipulate and observe neurons due to the fact that they maintain the main characteristic features of their tissue of origin, making them a biologically and physiologically relevant tool for the study of neuroscience. However, primary neurons are highly sensitive to isolation conditions and growth environment, do not proliferate, and were limited to short term culture (<5 days) [2]. On the other hand, neuronal cell lines are commonly used for in vitro neurobiology studies because they are easily transfected, possess the ability to proliferate, and can be induced to differentiate into neuron-like cells, expressing neuronal biomarkers and presenting axons and dendrites. For this, commercial immortal neuronal cell lines, which can simulate neurons after differentiation process are available: PC12, SH-SY5Y, bEnd.3, TR-BBB, HBMEC, BV2, etc. Based on the differentiation time (i.e., about 4 days for PC-12 and 18 for days for SH-SY5Y), and the neurogenic characteristics, PC12 is one of the most preferred cell line to be used as model in neurobiology studies [3].

In order to obtain detailed information on the mechanism of intra- or extra-cellular reactions involving cells under different stimuli, mimicking different pathophysiological processes, an appropriate analytical approach is needed. In this regard, the (bio)sensors field represent an excellent tool recognized for fast, low-cost, and innovative methodologies that can be designed for in situ analysis of cell cultures, improving in vitro models, thus allowing the study of molecular mechanisms to be carried out in a simple and reproducible manner [4]. This review provides an overview of the wide application of biosensing in PC-12 cell culture and their potential application values in understanding nervous system diseases.

## 2. PC-12 Cell Line

For over half a century, biosensors have been widely investigated and nowadays they are playing an essential role in various sectors such as food and pharmaceutical industry as well as in various medical fields. Integration of cells cultures within biosensors are common practices now and brings great value to the field of medical, pharmaceutical, and biological research.

Neurobiology is a field intensively studied with the help of cell-based biosensors. Few types of neuronal cells precursors are investigated and used with this experiments, Mouse Hypothalamic GnRH Neuronal Cell Line GT1-7, human neuroblasts (SH-SY5Y), pheochromocytoma cells (PC-12), just to name a few.

PC-12 cell line is preferred as model in neurobiology studies because of its neurogenic characteristics (after differentiation) such as neurotransmitters secretion (dopamine, norepinephrine, and other catecholamines), neuron morphology, as well as ion and neurotransmitter receptors.

Being extensively used, PC-12 cells come with the advantage of a great amount of knowledge about culturing conditions and differentiation process. In opposition to SH-SY5Y, PC-12 adh cells have the advantage of taking shorter time to differentiate.

**Figure 1:**
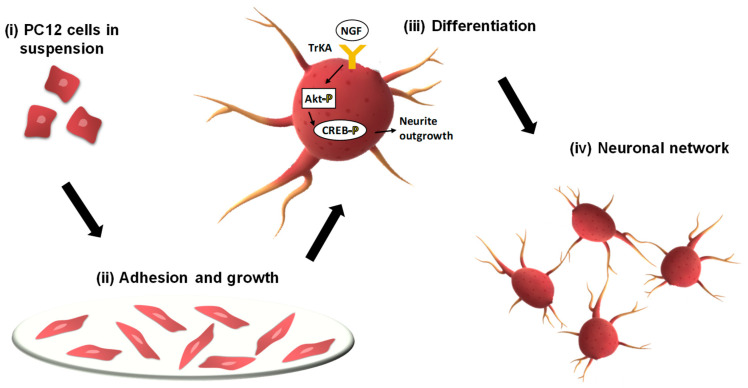
Schematic representation of PC-12 neuronal network formation.

PC-12 is harvested from a pheochromocytoma rat adrenal medulla and is one of the most common neuronal precursor cell lines used in neuroscience research including studying neuronal degenerations, neuronal differentiation, and neural networks. Cultured under normal conditions, PC-12 cells present morphological and physiological characteristics of the adrenal gland cells. When nerve growth factor (NGF) is added to the culture, this type of cells suffers a differentiation process and start to manifest morphological and functional characteristics of sympathetic ganglion neurons, Figure 1.

There are two types of PC-12 cell lines, classical PC-12 cells grown in suspension and the adherent line of PC-12 cells originated from selected phenotype of easily adherent cells. To increase the ability of PC-12 in suspension to become adherent it is necessarily to modify with collagen the surface on which there are cultured. Adherent line of PC-12 attaches easily on poly-d-lysine surface as well as on collagen and even on plastic surface [5].

Optimization of nerve growth factor (NGF) concentration added to the culture is of great importance for the differentiation process of PC-12. It has been observed that with the same concentration of NGF, cells in suspension reached full neuronal characteristics within 14 days of incubation and adherent line of PC-12 took 3 to 5 days of incubation to show proper differentiation after which they start to proliferate. This and the fact that PC-12 adherent line attach easily to several types of surfaces makes them a better model to use with cell-based biosensors application [6].

Several cell-based biosensors experiments were employed to investigate neuronal cell morphology, neurodegenerative chemical-induced effects, neuronal response to stimuli (K^+^, Zn^2+^, nicotine), intra and extracellular detection of several molecules (ions, neurotransmitters), and expression of neuronal biomarkers [7].

Research regarding synapse formation can be conducted on PC-12 after differentiation because of the capability to form functional synapses with each other. Culturing and differentiating PC-12 cells on microelectrodes set up the chance to study in real time neurological synapses formation and function in a noninvasive way, recording simultaneous extracellular potential of neuronal cells over long periods of time [8].

## 3. Biosensing and Biosensors

Biosensing represents the detection of target molecules (analytes) based on the chemical principles used by a living system, e.g., molecular recognition. A biosensor is a device that transforms chemical information into an analytically useful signal and contain two connected components: a receptor, consisting in a recognition system (antibody, enzyme, aptamer, cell, etc.,), and a physicochemical transducer. The biological recognition system translates the interaction between the analyte and receptor into a measurable signal transferred by the transducer to a measuring device. Biosensing represents the detection of target molecules (analytes) based on the chemical principles used by a living system, e.g., molecular recognition. A biosensor is a device that transforms chemical information into an analytically useful signal and contains two connected components: a receptor, consisting in a recognition system (antibody, enzyme, aptamer, cell, etc.,), and a physicochemical transducer. The biological recognition system translates the interaction between the analyte and receptor into a measurable signal transferred by the transducer to a measuring device [9].

According to the nature of its transducer and the detection method, a biosensor can be: piezoelectric, electrochemical, electronic, optical, thermal, etc. For biosensing in PC 12 cell culture, electrochemical (e.g., voltametric, amperometric or impedimetric), electronic (i.e., field effect transistor), and optical biosensors (e.g., fluorescent) are the most commonly used.

The electrochemical biosensor is one of the typical biosensing devices based on transducing the biochemical events to electrical signals. The transductor is represented by the working electrode, a key component that is employed as a solid support for immobilization of biomolecules and electron movement. More details regarding the electrochemical biosensors architectures and detection methods can be found in [10,11].

The field effect transistor is a type of transistor that uses an electric field to control the conductivity of a semiconductor material channel between two electrodes, i.e., the source and drain, while the control of the conductivity is achieved by varying the electric field potential, relative to the source and drain electrode, at a third electrode, known as the gate. More details regarding the characteristic features of FET biosensors are presented in [12,13].

A particular type of biosensor is cell-based biosensor, a device capable to connect viable cells (as sensing receptors) with transductors, having the purpose of intercepting intracellular and extracellular physical, chemical, and physiological signals, and releasing significant information about the interaction of cells with environmental stimulus [14]. The two principal elements of these biosensors consist of living cells (receiving and releasing signals in relation to the inner or external stimuli) cultured on the surface of the transducer (able to convert physical, physiological or chemical signals to electrical signals). Because of the ability to integrate living cells into the process of receiving and converting biomolecular species/interactions in electrical signals that can be measured and transposed into analytical information, cell-based biosensors bring tremendous advantage to several industries as pharmacology, clinical diagnostics, cell biology research [15].

Carbon-fiber microelectrodes [16] have proven to be a useful tool for monitoring exocytosis events using amperometry or voltammetry [17]. The quantification and analysis of vesicular content of metabolites, neurotransmitters, reactive oxygen and nitrogen species, glucose, oxygen, hydrogen peroxide, and ions in cells are important for studying the mechanism of neurotransmission [18,19].

Microelectrodes array (MEA) represents a matrix of microelectrodes fabricated on silicon or glass surface. It can be used to measure cell action potential and the main strength of MEA is the ability to evaluate electrophysiological activity of cell cultures, the capability to detect extracellular action potentials and neuronal signals to several substances making it an important tool in medical and pharmaceutical field [20,21].

Similarly, cell-based field effect transistor (FET) biosensors are able to transduce the cellular metabolism information expressed extracellular concentrating on the differences of ionic concentration at the gate area and at the cell membrane action potential. Cell-based FETs are widely used to measure ions as those from cell respiration and extracellular pH [21].

Nevertheless, properties of cell membranes from bioelectrical perspective, including extracellular potential signals released from the cells, electrostimulation, detection of cellular metabolism products, and screening of pharmaceutical compounds can be achieved by using other types of cell-based biosensors such as light addressable potentiometric and electric cell-substrate impedance sensors. The main characteristic features of cell-based biosensors were presented in [7,22].

Optical biosensors are also commonly used in several applications involving detections of small molecules such as drugs, ions, antibodies, biomarkers, parasites, and even entire cancerous cells. An optical biosensor is a device which combines a sensing reception part with an optical transducer and the main objective is to generate a response as a proportional signal to the concentration of the analyte [23,24].

## 4. Metal Ions and Small Molecules Detection

### 4.1. Metal Ions

Metal ions are involved in cellular and subcellular functions with many functions still unrevealed and with a demand to elucidate the role of inorganic salts in living systems.

**Figure 2:**
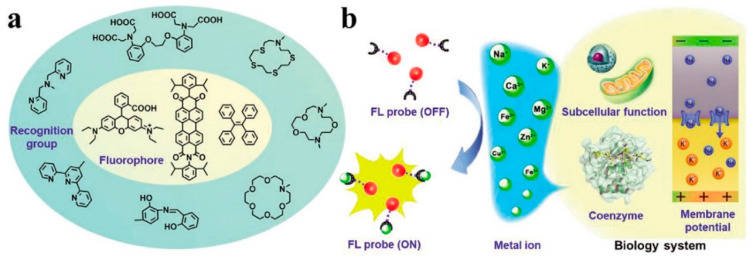
Schematic illustration of (**a**) the composition and sensing mechanism and (**b**) biological applications of metal ions probe. Reprint from [25] under Creative Commons Attribution 4.0 International License.

Some of the known functions of metal ions are: material transportation, energy conversion, information transmission, and metabolic regulation. Their low or high concentration can lead to health issues, so the detection and mapping of metal ions in individual cells, tissues, and whole organisms is crucial. Among the methods employed for metal ion detection, fluorescent sensors are the most applied ones since they also allow imaging of the metal ions in biological systems, such as Na^+^, K^+^, Ca^2+^, Mg^2+^, Fe^2+^/Fe^3+^, Zn^2+^, and Cu^2+^. A classic organic fluorescent probe contains one or more fluorescent cores, a metal chelating or binding moiety which is able to recognize and interact with different metal ions, and their use in the sensing mechanism for biological applications in metal detection is illustrated in Figure 2 [25].

Several metal ions have been successfully quantified or imaged in PC-12 cells, bringing important insights into their physiological and pathological role, and are listed below.

Zinc ions are mostly known to play an important role in signaling in both intra- and intercellular communication, which require transients of free zinc ions, and not protein-bounded zinc. This represents a challenge since it is quite difficult to distinguish between the two forms of free and bounded zinc. For this purpose, two excitation ratiometric fluorescent biosensors based on carbonic anhydrase were reported [26,27]. No interferences form calcium or magnesium ions in millimolar concentration range were observed, which allowed to detect zinc ions in a very low concentration range of 5–10 pM in cytoplasm and nucleus [26]. The detection of free zinc level variation upon cellular oxygen glucose deprivation (OGD) in cytoplasm and mitochondria in the PC-12 rat pheochromacytoma cell culture line enabled to determine that there is an increase in the mitochondrial free zinc ions concentration immediately after OGD, which than gradually returned to physiological levels, while cytosolic zinc increased over a 24-h time period in viable cells. The findings are valuable to better understand bioenergetics dysfunction and cell death that occurs with both in vitro and in vivo models of reperfusion [27].

Copper is a required trace element with important biological roles, being a component of several human enzymes, making cupper deficiency or defects in copper transport leading to serious or fatal diseases. The use of a synthetic variant of human apocarbonic anhydrase II for sensing Cu^2+^ enabled and increased selectivity for Cu^2+^ over Zn^2+^. The biosensor based on the fluorescent-labeled Cu^2+^-specific variant of human apocarbonic anhydrase was able to measure very low concentrations of Cu^2+^ in the femtomolar range in PC-12 cell cultures with the possibility to also image the free Cu^2+^ levels by means of frequency-domain fluorescence lifetime microscopy [28].

Calcium ions were reported to be the key analyte for various events comprehensions in PC-12 cells, especially in synaptic transmission and spontaneous neurotransmitter release [29,30,31]. Single cell-based biosensors were also reported for calcium ion concentration detection by using fluorescence microscopy with the ion indicator fluo-3-acetoxymethyl ester to measure receptor activation in PC-12 lines [32] and for the identification of biologically active ligands present in a complex mixture. In the first case, the single-cell biosensor was based on the ligand–receptor binding and G-protein-mediated signal transduction with applicability in the identification of endogenous bradykinin, a peptide that promotes inflammation, in human hepatocellular carcinoma cells lysates and its bioactivity screening in degradation products from human blood plasma [32]. The second biosensor was able to detect specific components of a complex mixture fractionated by a microcolumn separation technique based on ligand–receptor binding and signal-transduction pathways to biochemically amplify the presence of an analyte after electrophoretic separation [33]. Another biosensor used the Ca^2+^ enhancement of the novel synthetized coumarine derivative fluorescence and a detection limit of 5.81 × 10^−7^ M, for calcium, was obtained [34].

### 4.2. Neurotransmitters

Neurotransmitters are small molecules that act as messengers in the synaptic transmission process, with imbalance in their activity causing serious mental disorders, such as Parkinson’s disease, schizophrenia, and Alzheimer’s disease. Therefore, monitoring the neurotransmitters concentrations is of great interest in the study and diagnosis of several mental illnesses. Biosensors for in vivo and ex vivo neurotransmitter detection rely on the use of nanomaterials, polymers, and biomolecule, with the electrochemical ones prevailing in the in vivo detection over the optical ones [35,36].

Exocytosis plays an essential role in the communication between cells in the nervous system. Understanding the regulation of neurotransmitter release during exocytosis and the amount of neurotransmitter content that is stored in vesicles is of importance, as it provides fundamental insights to understand how the brain works and how neurons elicit a certain behavior [30,37].

Catecholamines are a class of neurotransmitters comprising dopamine (DA), norepinephrine, and epinephrine. The correlation of catecholamine levels with many types of diseases, motivated many researchers to develop accurate quantification methodologies together with optimization procedures and sample preparation to detect them at extremely low concentrations in the presence of numerous co-existing biological interferences [38].

Among the catecholamines, DA plays a key role in the function of the human central nervous system. Since abnormal release of dopamine is associated with several neurological diseases, its detection and continuous monitoring in vivo has been of great interest, with many electrochemical and optical biosensors being developed for this purpose. The main focus was to achieve low detection limits, due to the fact that concentration of DA is extremely low in patients with neurological diseases [39]. In this context, PC-12 cell lines play an important role in the detection of DA in vivo, with several optical [40,41,42], electrochemical biosensing systems, fixed potential amperometry [43,44,45,46,47,48,49,50,51] and voltametric ones [52,53,54], and electrical devices [55,56] being investigated, with great potential for diagnostic purposes being demonstrated.

Catecholamine release from PC-12 cells has been observed at zeptomole levels using dc-amperometric detection at carbon fiber microelectrodes. The results obtained from 13 PC-12 cells corresponded to 190 zmol (114,300 molecules per release event) and the detection limit was as low as 31 zmol [57]. Moreover, cyclic voltametric measurements of relative concentration for zeptomole levels of transmitter in attoliter volumes provide evidence that loading vesicles by increased transmitter synthesis does not lead to elevated concentrations at individual release sites [58].

Structural and size analysis of the vesicular dense core and halo using transmission electron microscopy was combined with single-cell amperometry to study the vesicle size changes induced after zinc treatment, and the existence of a strong link between vesicle structure and exocytotic dynamics was established [59].

Several electrochemical biosensors were reported for DA detection in PC-12 cells, most of them using fixed potential amperometry [43,44,45,46,47,48,49,50,51] and voltammetry [52,53,54].

The amperometric biosensors operated at similar voltage values of around 0.3 V, normally chosen after evaluating the voltametric profile of DA at the corresponding developed sensing platform. The biosensor based on Au microelectrodes modified with conductive polymers and peptide nanofibers allowed the amperometrical detection of DA in the femtomole range, having the advantage in using the peptide nanofibers that increase the adherence properties of PC-12 cells [43]. A nitrogen-doped mesoporous carbon nanosheets-based biosensor operated at +0.25 V and showed high sensitivity and selectivity for DA sensing with a detection limit of 10 nM [44], with a similar detection limit value of 9 nM achieved by a paper-based electrochemical sensor [46] and a slightly lower value of 5 nM by the one based on graphene oxide and AuNP with EDTA immobilized-poly(1,5-diaminonaphthalne) [45]. The use of a nanocomposite comprising Pt nanoparticles (PtNPs) decorated multi-wall carbon nanotubes (MWCNTs), allowed the lowest detection limit among the amperometric biosensors, with a value of 2 nM [47]. The release of neurotransmitters from PC12 cells under stimulation was selectively monitored by amperometry at carbon fiber electrode in order to detect either solely dopamine or dopamine and FFN102 altogether. It was observed that FFN102 led to the partial replacement of dopamine in secretory vesicles and the partial replacement of dopamine could be used to monitor exocytic events through electrochemical detection of dopamine and fluorescence detection of FFN102 [60].

The development of electrochemical cytometry allows comparison between vesicle content and vesicular release and it was found that only part of the vesicle content is released in typical exocytotic cases measured by amperometry. The approach involves the adsorption and subsequent rupture of vesicles on an electrode surface following electrode redox processes. The measured current allows to count the number of molecules in the vesicles using Faraday’s law and to correlate this to the amount of molecules released when single exocytosis events take place in communicating cells [61].

Other amperometric arrays use a microfluidic sensor based on carbon nanotubes (CNTs)-modified indium tin oxide (ITO) microelectrodes for DA detection in single living rat pheochromocytoma (PC 12) cells [48], while the microdialysis-amperometric system reported in [49] uses high-performance liquid chromatography with electrochemical detection (HPLC–EC) [49], and the scale-integrated amperometric sensor in [50] was able to image simultaneously the DA release from PC-12 cells. Besides the amperometric detection assays exemplified above, which operated at relatively low overvoltage of around +0.3 V, another assay based on micro graphitic-diamond multi electrode arrays was applied for the detection of DA released form PC-12 cells under K^+^ stimulation at a higher overvoltage of +0.65 V [51].

One voltametric biosensor uses a 5-nm-thick poly-celestine blue (CB)-modified glassy carbon electrode (GCE), with a detection limit of 1.2 nM with successful application for DA detection released from PC-12 lines under nicotine stimulation [52], while the other is based on molecularly imprinted layers of 4-mercaptophenylboronic acid (4-MPBA) and the polymer acid chrome blue K, which is polymerized around the DA molecule to achieve high selectivity toward DA. The later sensor had a detection limit of 140 nM [53]. The lowest detection limit of voltametric biosensors was achieved by a graphene quantum dots/multiwalled carbon nanotubes (GQDs-MWCNTs)-based biosensor with excellent selectivity for DA and a detection limit of 0.87 nM using DPV [54].

Almost all electrochemical biosensors were successfully applied for DA detection released from PC-12 cells under K^+^ [44,45,47,50,51,53] or nicotine [52] stimulation. An important applicability was achieved by the amperometric biosensor in [47] which was able to evaluate the effects of antipsychotic drug (aripiprazole) on the dopamine release from cells treated with high K^+^ and by the paper-based one in [46] which monitored the DA released from damaged PC12 cells induced by amyloid-beta peptide (Aβ_25–35_) and cell intervene models protected by curcumin and marrow mesenchymal stem cells. The sensing platform developed in [49] also showed an important applicability in studying the effects of different drugs on DA secretion with the device in [50] exemplifying the effects of the dopaminergic drugs l-3,4-dihydroxyphenylalanine (l-DOPA) on the DA levels released form the PC-12 cells.

Lower values of detection limits toward DA detection were achieved using field effect transistors (FET) [55,56], one of them based on DNA-aptamers immobilized on a multiple-parallel-connected silicon nanowire (SiNW) with a detection limit lower than 10 fM, with high specificity for DA in the presence of ascorbic acid, catechol, phenethylamine, tyrosine, epinephrine, and norepinephrine. The FET enabled the DA monitoring released from PC-12 cells under hypoxic stimulation which is coupled to an increase in intracellular Ca^2+^ that is required to trigger DA secretion [55]. Another FET based on SiNW was developed using the chemical linker 3-aminopropyltrimethoxysilane (APTMS) and boronic acid, with high binding affinity achieved and a dissociation constant of dopamine–boronic acid complexes of 33 ± 8 fM [56].

Besides DA, epinephrine and norepinephrine were also the key analytes to be detected by several newly developed biosensing systems, with in vivo detection in PC-12 cells under various external stimuli.

Norepinephine was detected electrochemically in PC-12 cells on electrode platforms based on C-, N-doped NiO, which make use of both the homogenous doping and the nanostructure NiO surface to enhance the electroactivity of the sensor toward a sensitive noradrenaline detection released form PC-12 cells under K^+^ extracellular stimulation, with high selectivity and long-term stability [62]. Another electrochemical sensor for norepinephrine detection released from PC-12 cells under K^+^ stimulation was based on nitrogen-doped carbon hollow trunk-like structure which demonstrated high selectivity for monitoring of NA with a detection limit of 5 nM. Furthermore, this sensor is a portable sensor, an advantage for POC applications [63].

The neurotransmitter epinephrine is also a well-known medication in resuscitation, especially after a heart attack and to treat bronchial asthma attacks. Hence, its detection is crucial for the discovery and evaluation of new epinephrine-based drugs to control the metabolic processes linked to diseases, with applicability in the POC devices. Epinephrine was colorimetrically and electrochemically detected in PC-12 cells at CuO nanorods with laccase-mimicking properties. The dual biosensor had no interferences from dopamine, ascorbic acid, and uric acid, with detection limits of 0.31 μM and 20 nM for the colorimetric and voltametric method, respectively, underlying the advantage in using the electrochemical technique for epinephrine detection at very low concentrations [64].

Besides the above mentioned catecholamines, glutamate plays an important role in the excitatory neurotransmission in the mammalian brain, and its fast removal from the synaptic cleft is critical for preventing toxicity and spillover to neighboring synapses. Its detection in PC-12 cells was achieved using a fluorescent indicator protein for glutamate from *Escherichia coli*, which in the presence of ligands, has a concentration-dependent decrease in the fluorescence signal. Consequently, the depolarization of neurons leads to a fluorescence signal decrease corresponding to 300 nM glutamate at the cell surface, with no change in the signal when cells were exposed to 20 mM glutamate, the minimal glutamate uptake in the cytosol [65].

In addition to neurotransmitters, hydrogen peroxide release plays an important physiological role in cell-to-cell signal transduction, and amperometric detection at gold, platinum, and/or graphene transductors is generally preferred. Using a three-dimensional nanoporous gold electrode decorated with ultra-thin platinum nanoparticles, a wide linear range, from 0.05 μM till 7.37 mM, for the amperometric determination of H_2_O_2_, at an applied potential of −0.4 V, was obtained [49]. A limit of detection of 1.5  ×  10^−8^ mol/L and a high sensitivity of 1.125 μAμM^−1^ cm^−2^ were achieved. Nevertheless, the biosensor has been applied to the dynamic determination of H_2_O_2_ released from PC-12 cells and a value of 52.5 amol H_2_O_2_, generated by each cell, was obtained [49].

**Figure 3:**
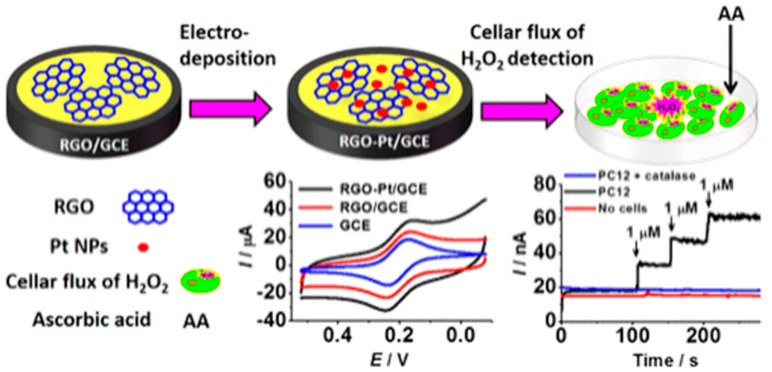
Schematic representation of reduced graphene oxide-platinum nanocomposite-modified glassy carbon electrode and the H_2_O_2_ detection in PC-12 cell culture. Reprint with permission from [66]. Copyright (2014) American Chemical Society.

Similar linear range was obtained at a reduced graphene oxide-platinum nanocomposite-modified glassy carbon electrode, Figure 3 [66]. Although the detection limit was higher, 0.2 μM, the biosensor allowed the detection of H_2_O_2_ release from living cells.

Fluorimetric sensors were also reported, one based on BSA-stabilized Au nanoclusters [40] and one based on single-walled carbon nanotubes (SWCNT) [41]. In the first case, the detection principle is based on the decrease of the fluorescence intensity emitted by the sensor upon the attachment of DA to BSA which leads to a photo-induced electron transfer process. The sensor exhibited a detection limit of 10 nM, with no interferences form possible interfering substances and successful application for the DA monitoring in PC-12 cells [40]. In the second methodology, arrays of fluorescent nanosensors based on SWCNT are placed under and around neuroprogenitor cells to enable the imaging of the released DA from the PC-12 cells following K^+^ stimulation. The acquired fluorescence map revealed areas where DA is released on the cell’s surface and how its morphology affects the location of release sites. Moreover, the results elucidate how membrane morphology influences the directionality of chemical signaling by DA [41].

Another optical biosensor based on three-dimensional tungsten disulfide (WS_2_) was reported using Raman spectroscopy detection. The sensor uses 2D WS_2_ directly grown on a 3D WO_3_NH by sulfurization, with advantages over the 2D support array of WO_3_NH on the adsorption of biomolecules and cells proliferation which enhances sensor sensitivity to DA [42].

Combining the lithography and electrochemistry method, a metal-free graphene based hybrid microelectrode array for sensitive and in-situ amperometric sensing of H_2_O_2_ was fabricated and a detection limit of 0.18 μM, in PC-12 cell culture, was achieved [67].

## 5. Cellular Events

### 5.1. Attachment, Proliferation, and Differentiation

The main characteristics of PC-12 as a neuronal cell line is the ability to develop neurite outgrowths during the differentiation process; a process that demands the cells to be attached on a surface. Usually, the PC-12 cells grow as small, irregularly shaped cells, adherent or in suspension, floating in the growth media, and tend to form aggregate and adhere poorly to non-coated surfaces. Therefore, the control of neuronal cell patterning needs modified surfaces commonly coated with fibronectin, laminin, poly-L-lysine, collagen, etc.

For neurite growth guiding, collagen-coated electrospun gelatin/polycaprolactone nanofiber mats on microstructured polystyrene surface were used obtaining an increased adhesion, differentiation, and guided neurite outgrowth compared to controls [68].

Another approach for increasing cell adhesion is to take advantage of the physicochemical properties of the of electroconductive hydrogels. This showed integration of electropolymerized polypyrrole networks within poly(hydroxyethylmethacrylate)-based hydrogels and controlled the elastic modulus and the electrical impedance properties of electroconductive hydrogels [69]. Growth of attachment-dependent PC-12 cells at the hydrogel showed to be dependent with electropolymerization charge density [69].

Quartz crystal microbalance (QCM) has been applied to develop cell-based biosensors as secondary sensors to deliver functional information of cells such as the cell attachment, proliferation, and cell–substrate interaction under different conditions. QCM was used to monitor the mass change and rigidity of populations of excitable cells during exocytosis and subsequent retrieval of dense-core vesicles and it was observed that stimulating the cells to exocytosis with elevated potassium concentration resulted in an increase in the frequency response corresponding to loss of mass from the cells owing to release of vesicles [70].

Optical biosensors based on surface plasmon resonance (SPR) have the potential for investigations of cell responses and real-time monitoring of individual cell responses to various exogenous substances under ambient conditions. This technology addresses cell monolayers cultivated on the gold sensor chip and allows the evaluation of compound potency, specificity, selectivity, toxicity, and effectiveness at the level of individual cell. Thus, based on reflection intensity changes of SPR, the intracellular translocation of protein kinase C (PKC) of PC 12 during differentiation process was observed [71,72], and the detection of neuronal differentiation in live cells, at the level of individual cells, was achieved [71]. Moreover, a high sensitivity and enhancement of SPR response to muscarine stimulation was found for the cells treated with the nerve growth factor [71].

Live cell-fluorescent biosensors allow the collection of temporal information about cellular events, from changes of the membrane potential of the cell till cell cycle progression and arrest. Measuring the changes of cell membrane potential, the level of superoxide anion generated during the in vitro differentiation of PC-12 cells was determined in a noninvasive way [73]. Nevertheless, using a fluorescent biosensor, it was demonstrated that NGF-induced neurite extension occurs independently of NGF-induced cell cycle G1 phase arrest. This finding allowed the investigation of the PC-12 proliferation at the resolution of individual cells and neuronal differentiation as a dynamic process of parallel cell cycle arrest and neurite outgrowth [74].

The axonal elongation requires axonal membrane growth by exocytosis of plasmalemmal precursor vesicles at the nerve growth cone, a process regulated by nucleotide guanosine triphosphate (GTP) hydrolase enzymes family [65]. Similar to fluorescence, Forster resonance energy transfer (FRET)-based biosensors allow the acquisition of valuable information about cellular events such as exocytosis, neurite outgrowth, proliferation or differentiation, as well as the biochemical mechanisms involved. Using a FRET biosensor it was showed that the activity of TC10, a GTP enzyme, at the plasma membrane decreased at extending growth cones in NGF-treated PC12 and it was demonstrated that cells signaling machinery containing TC10 is used for exocytosis [75].

Light-addressable potentiometric sensor (LAPS) allows the fabrication of interfaces between the physical and biological system, i.e., semiconductor—biological cell. To improve the biological cell adhesion, the LAPS insulator usually is coated with biocompatible molecules. Thus, efficient culturing of PC-12 on 4 nm poly-l-ornithine and laminin layer coated LAPS structure was achieved [76]. This layer enhanced sensitivity and allowed simulation of neural action potential applied to the LAPS [51].

Impedance biosensors are very useful for real-time monitoring of extracellular matrix-mediated PC12 cell attachment and proliferation [77], adhesion and differentiation [78,79]. The attachment and proliferation of the neuron-like cell line PC-12, on different extracellular matrices, confirmed by MTT assays and a scanning electron microscopy analysis, was monitored using cellular impedance sensing [77]. Similar, using interdigitate microelectrodes array, the attachment, differentiation, and formation of synapse-like contacts between the neuronal cell and the conductive surface of a microelectrode array were followed by recording changes of impedance [78,79] and it was demonstrated that the complex impedance is dependent on ion fluxes at the neuron-to-electrode contact surface.

**Figure 4:**
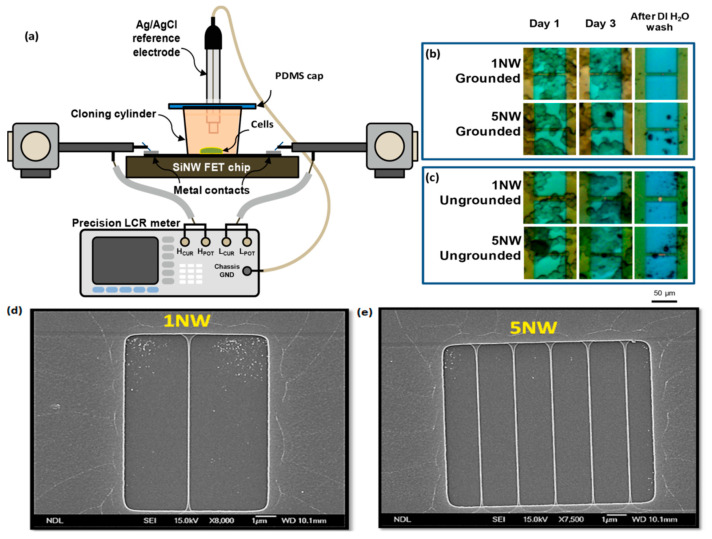
(**a**) Impedance measurement setup for monitoring cell-coupled p-type SiNW FET devices with the reference electrode was connected to the chassis ground of the LCR meter. Microscopy images of cell coupled on 1NW and 5NW FET devices with grounded and ungrounded setups on days 1 and 3 of culture and after DI water wash were shown in (**b**) and (**c**), respectively. Accordingly, SEM images of 1NW and 5NW FET devices are shown in (**d**) and (**e**). Reprinted with permission from [80]. Copyright (2022) Elsevier.

Cell-coupled silicon nanowire field-effect transistor devices were exploited to elucidate the effect of cell proliferation on impedance spectra, Figure 4. Owing to the hindrance of ions or negatively charged membrane of the coupled cells, the changes of carrier density within transistors correspond to the signals in impedance spectra and can be used to probe cell condition during the growth [80].

### 5.2. Action Potential Cell Stimulation and Intracellular Signal Transduction

Real-time observation of intracellular process of signal transduction is very useful for biomedical and pharmaceutical applications as well as for basic research work of cell biology. For feasible and reagentless observation of intracellular alterations in real time, a SPR biosensor was applied in PC-12 cells culture for monitoring of intracellular signal transduction that was mainly translocation of protein kinase C. This was achieved via local refractive index change in PC-12 cells adhered on a gold sensor slide after stimulation with KCl and phorbol-12-myristate-13-acetate (a protein kinase C activator), at different concentrations, in order to induce intracellular PKC translocation [81]. In another study, similar achievements were obtained using a nonadiabatic tapered optical fiber biosensor [82].

The stimulation of PC-12 cells with epidermal growth factor (EGF) leads to transient extracellular signal-regulated kinase (ERK) activity and cell proliferation, whereas nerve growth factor (NGF) stimulation leads to sustained ERK activity and differentiation. Using a FRET-based biosensors it was showed that both NGF and EGF potently activate PKA at the plasma membrane, although they generate temporally distinct activity patterns [83].

Coupling an ultra-thin microelectrode array with total internal reflection fluorescence microscopy, simultaneous recording of action potentials and neurotransmitter release was obtained. The combination of the optical and electrical techniques enabled mapping of neuron connectivity in an entire neuronal circuit. Moreover, the real-time recording of action potential and neurotransmitter release reveals the relevance of electrical and chemical activities in the neuronal mode [8].

The real-time electrochemical investigation of PC-12 cell culture was accomplishing using a membrane-based electrode sensor. The electrochemical performance of the membrane electrodes was characterized by cyclic voltammetry and chronoamperometry, and the detection of synthetic dopamine and dopamine exocytosis was demonstrated down to a concentration of 3.1 pM [84].

Microelectrode arrays, composed of 60 independent electrodes, for stimulation and signal recording of in vitro cultured neurons were coupled with impedance measurements [85,86]. The extracellular signal recording in the presence of acetylcholine and other stimulants has been carried out and the results indicate that microelectrodes array systems can be used for extracellular stimulation, recording, simultaneous stimulation and recording, and isolation of PC12 cells network cultured in vitro [85,87].

Monitoring of electrophysiological properties of cultured neuron networks derived from PC-12 was achieved using a neurochip based on LAPS biosensor technology. After the differentiation of PC-12 cells and formation of neuronal networks on LAPS system, the extracellular potentials were recorded. The results showed that the neurochip of PC-12 cells coupled to LAPS is stable and suitable for long-term and non-invasive measurement of cell electrophysiological properties [88].

### 5.3. Monitorization of Cytotoxic Effect

Fast and selective monitoring of dopamine release is essential for screening pharmaceuticals that may be beneficial in the treatment of catecholamine-related psychiatric disorders [89]. The complexity of the exocytosis process, dopamine re-uptake, diffusion, and auto-oxidation are crucial parameters when employing biosensing methodologies, as well as considering the pharmaceutical’s distinct mechanism of action over dopaminergic pathways, which may involve inhibition of vesicular monoamine transport (VMAT), dopamine transport (DAT), catechol-O-methyltransferase pathway, dopamine precursor supply, and activation of dopamine receptors. In this context, the development of a rapid electrochemical approach has been reported toward screening of pharmaceuticals targeting dopamine-related psychiatric disorders. For this, microelectrode array biochips based on gold covered silicon wafers were used to investigate the effect of three drugs (L-dopa, reserpine and nomifensine) with different mechanisms of action upon dopamine release, the latter monitored using chronoamperometry upon K^+^-stimulation of drug-treated PC-12 cells [90]. A two-cell-based biosensor was also developed to monitor the influence of receptor antagonists (ATP, acetylcholine), K^+^-induced membrane polarization, and neurotoxins (black widow spider venom, α-latrotoxin) on neurosecretory output of PC-12 cells. For this, the transducing mechanism was achieved through optical monitoring changes in naturally colored fish chromatophores, where varying the catecholamine secretion level under stimuli led to pigment aggregation [91].

It is clear that when dealing with living cell assays, the biocompatibility of the sensor materials is of utmost importance to support a good environment for cell adhesion and proliferation, whilst avoiding toxicity which may be induced by the material itself, such as reported for dose-dependent exocytosis induced by silver nanoparticles [92]. Some strategies describe the use of paper-based scaffolds to improve cell implantation, thus maintaining a three-dimensional similarity to the cell physiological microenvironment. As an example, a 3D-based cell culture system based on paper-polylactic platform coupled with an electrochemical sensor surface was used to monitor cell damage upon interaction with amyloid-beta oligomers. The excellent biocompatibility of the materials as well as efficient cell implantation supported the investigation of the cytoprotective effects of donepezil and BMSCs-secreted active molecules upon the inflicted cell damage [93]. A different approach employed a 250 μm gold microelectrode fabricated within cell culture biochips, whilst surface attachment of PC-12 cells was conducted using self-assembled monolayers of cysteamine covalently derivatized with laminin. In this study, electrochemical impedance spectroscopy was used to characterize Ca^2+^ exocytosis fluctuations upon treatment of living cells with calcimycin, nifedipine, mannitol, and carbachol, as well as to correlate phenotypic alterations due to cell exposure to nerve growth factor (NGF), dexamethasone, and forskolin [94]. Similar, field-effect transistors based on semiconducting single-walled carbon nanotubes were successfully used for monitoring the effects of histamines on Ca^2+^ release from the intracellular stores [95].

Neurotoxins are often destructive compounds able to inflict damage upon nerve tissue, often leading to neuron excitotoxicity or apoptosis. The influence of rotenone, okadaic acid and peroxynitrite, neurotoxins that induce cell death on PC-12 cell lines, has been evaluated with electrochemical impedance spectroscopy at a cell-based array of gold electrodes complemented by artificial neural networks (ANNs), able to discriminate against false positives [96]. Another approach was used to electrochemically monitor bisphenol and its analogues, environmental pollutants that have been reported to inflict adverse neurodevelopment on animals and humans through interaction with O-GlcNAcase, an enzyme involved in the control of neuronal functions [97].

The cell response to nuclear growth factor, which may induce differentiation, has shown to be dependent on different parameters including ion-exchange inhibition, as reported using a silicon-based cytosensor for continuous and real-time monitoring of extracellular acidification rate changes on living cells [98]. Monitoring neuronal differentiation has also been achieved for 2D SPR-based sensors, where the increase in refractive index has been observed for differentiated cells stimulated with muscarine and carbacol [99]. Another approach investigated the intracellular signal transduction upon carbacol stimulation, whilst the reflection intensity change response has been suppressed by different natural compounds [100]. Finally, a high-throughput screening for small molecules able to bind on amyloid-β was conducted in order to identify natural inhibitors against Alzheimer’s disease. For this, biolayer interferometry together with an ultra-high-performance liquid chromatography coupled with diode-array detector and quadrupole/time-of-flight tandem mass spectrometry was used to assess the inhibition of amyloid-β fibrillation by polyporenic acid C, dehydrotumulosic acid, and tumulosid acid. Moreover, an improvement was observed in the viability of the amyloid-β-treated PC-12 cells [101].

### 5.4. Cellular Transport

Cell transport refers to the movement of neurotransmitters, ions, proteins, and other species through the cell membrane; the latter ensures cell structure and protection for the cytosolic content [102]. The importance on the membrane passive/active involvement to sustain cell homeostasis is considered a target for drug development and medical therapy, where, for example, K^+^, Ca^2+^ influx can be modulated, whilst catecholamine and cytokine release can be monitored to investigate the effects of such external stimuli upon the living cell environment.

**Figure 5:**
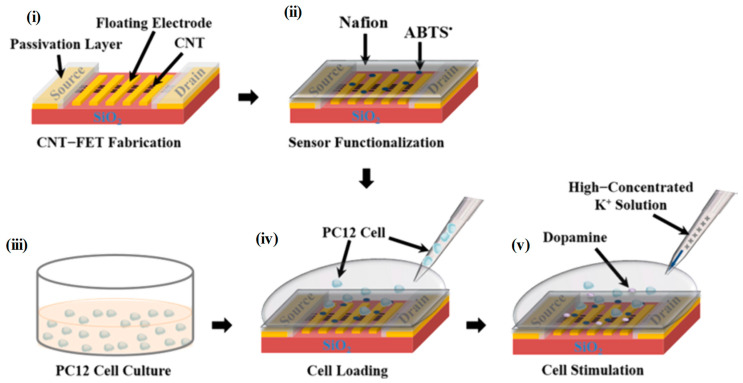
Schematic diagram depicting preparatory processes for the detection of dopamine by using an NRC sensor: (**i**) Fabrication of a conventional CNT-FET device with floating electrodes; (**ii**) functionalization of the device by a Nafion-radical hybrid film to build an NRC sensor; (**iii**) culturing of PC12 cells in RPMI 1640 medium; (**iv**) transferring of the cells into the sensor surface; (**v**) stimulating of the cells by a high concentrated K+ solution and measuring of dopamine release by using the sensor. Reprint with permission from [103]. Copyright (2019) American Chemical Society.

As previously mentioned, K^+^-evoked dopamine release is important to understand overall dopaminergic function. For this, a carbon nanotube (CNT)-based field effect transistor (FET) has been employed to monitor catecholamine release upon activation of membrane K^+^ ion-channels through K^+^ stimulation, as well as through incorporation of pimozide, an antipsychotic drug. The CNT-FET developed device was coated by a Nafion^®^ -radical hybrid film containing 2,2′-azino-bis(3-ethylbenzothiazoline-6-sulfonic acid (ABTS) radicals, Figure 5, thus ensuring the selectivity for dopamine detection up to 10 nM even in the presence of neurotransmitter interferents [103].

The maxi-anion channel is found in almost every part of the body, and is responsible for the control of membrane potential and biomolecules movement. The channel is activated in response to a variety of factors, ranging from osmotic cell swelling to physiologically and pathophysiologically relevant stimuli. In this context, a PC-12 cell-based biosensor was employed to investigate the special distribution of maxi-anion channels activity of a single rat cardiomyocyte through a patch clamp technique with scanning ion conductance microscopy. For this, local ATP release was monitored at concentrations of 1 mM through the PC-12 P2X receptor channels at a holding potential of −50 mV, whilst changing the placing of the biosensor in different areas of the cardiomyocyte. The special distribution of maxi-anions was assessed with fine-tipped pipettes, and results showed that they were concentrated at T-Tubules along Z-lines in adult cardiomyocytes. This approach further illustrated how the smart-patch technique can provide information regarding localized unitary events for specific transport channels, as well as for physiological/pathophysiological cell signaling investigation [104].

### 5.5. Cancer Cell Detection

The biosensing field has been a sophisticated alternative for early cancer detection, mainly due to vast versatility in constructions for selectively monitoring specific biomarkers, such as proteins or other biomolecules, often correlated to cellular malignancies. An alternative approach regards the early detection of cellular malignancies through the direct recognition of Jurcat, HeLa, PC-12, MDA-MB-231, and MCF7 cancerous cell lines as models for leukemia, cervical, adrenal gland, and breast cancer (type-1 and type-2), respectively.

The increased amount of proteins in cancerous cells leads to an increase of 1.37 to 1.40 in their refractive index, a parameter that can be easily monitored through optical biosensors for early detection of cancer. One approach described the use of 2D materials-based fiber-optic surface plasmon resonance (SPR) to monitor the angular shift and power loss of cancerous cells, thus distinguished from healthy cell lines [105]. Incorporation of different allotropes such as SnSe alongside a heterostructure of Blue P/MOS_2_ has also been shown to support the increase in refractive index variations to include monitoring cancer cells directly in biological solutions, as reported for the detection of skin, cervical, blood, adrenal gland, and breast (type-1 and type-2) cancer with a maximum quality factor of 24.83 RIU^−1^ and detection accuracy of 0.54 deg^−1^ [106]. In order to decrease measurement errors when compared with single-read biosensors, another approach was reported to monitor similar cancerous cells (including PC-12) through a portable micro-structured plasmonic based on a three-hole wagon wheel design [107].

Photonic crystals are also interesting platforms for the development of refractive index-based sensors, especially those used to monitor cancer cells [108]. One methodology described the infiltration of cancerous cells within defects between graphene and nanocomposite layers present in a one-dimensional photonic crystal biosensor, whilst their recognition was achieved through monitoring resonance peaks located within the transmittance spectrum [109]. Another approach involved the development of an ultra-compact biochemical sensor based on 2D photonic crystal cavity to monitor PC-12 cancerous cell lines [110]. Incorporation of gold nanowires as plasmonic materials has been reported to obtain improved performance of such optical biosensors for the recognition of MCF-7 and PC-12 cells with great sensitivity and resolution [111]. Photonic crystal fibers (PCF) also have been used to monitor cancerous cells, whilst the maximum values of sensing parameters were achieved for recognition of breast cancer (MCF-7) [112]. A polyamide substrate was also employed for immobilization of hexagonal gold layers, whilst an elevated yield confinement ensured a high sensitivity for monitoring basal, breast, cervical, and adrenal cancerous cells, the analysis being carried out using the full vectoral finite element methodology [113].

In light of the necessity to maintain sensitivity but also to improve the overall cancer detection in more point-of-care testing environment, a novel paper-based electrochemiluminescence (ECL) approach was reported for the detection of leukemia, through the use of a labelled aptamer specific to HL-60 cells. In the presence of cancerous cells, the aptamer would conjugate with glycoproteins at the cell surface, followed by the release in the label, rendering a decline in the ECL signal. This methodology, which was able to detect HL-60 cancer cells down to 56 cells/mL supports how paper-based approaches can still be tailored in point-of-care testing environment to monitor PC-12 cell lines for early adrenal cancer detection [114].

## 6. Conclusions

The immortalized PC-12 cell line demonstrated to be a classical neuronal cell model derived from rat pheochromocytoma with the ability to acquire the sympathetic neurons features in a differentiation process in the presence of nerve growth factor. PC-12 cell line was shown to be the preferred model in neurobiology study using biosensing devices and the analytical achievements and applicability of reported biosensing devices in PC-12 cultures for the detection of ions, neurotransmitters, and cellular events was summarized in this review. Nevertheless, a multitude of successful sensing applications using PC-12 have been demonstrated, many of which are mentioned in this review. The continued development of sensors and biosensors that address the detection of metal ions, neurotransmitters, as well as the evaluation of cytotoxic effect of different drugs and cellular events will substantially improve actual diagnostics and therapeutics methodologies.
